# Oto-tricho-tussia: An Unexpected Cause of Cough

**DOI:** 10.1155/2020/3527481

**Published:** 2020-03-31

**Authors:** Rebecca A. Castro, Craig H. Zalvan, Craig Berzofsky

**Affiliations:** ^1^Department of Otolaryngology, New York Medical College, Valhalla, NY, USA; ^2^The Institute for Voice and Swallowing Disorders, Phelps Hospital, Sleepy Hollow, NY, USA

## Abstract

Chronic cough is a frequently encountered condition with multiple etiologies. In patients with neurogenic chronic cough, peripheral laryngopharyngeal hypersensitivity of the vagus nerve stimulates the cough reflex. We present three cases of “Oto-tricho-tussia,” describing hair within the ear canal stimulating Arnold's branch of the vagus nerve and triggering the urge-to-cough. All three patients experienced significant improvement or complete resolution of their cough symptoms after removal of the hair resting on their tympanic membrane and external auditory canal. We encourage ear canal examination and promotion of proper ear cleaning habits as this is an easily treatable consideration for the cause of chronic cough.

## 1. Introduction

Oto (ear), Tricho (hair), Tussia (cough)—The otolaryngology community has seen an enormous increase in the number of office visits for chronic cough. In the 1980s, allergy was popularized as a common cause. The 90s saw a transition to reflux as the proposed dominant etiology. Over the last decade, we have seen a significant rise in the understanding of vagal neuropathy in the form of a neurogenic chronic cough (NCC) as the principle cause in patients with refractory chronic cough [1]. Cough plays an important physiologic role in expelling mucus and foreign bodies and noxious exposures, protecting the lower respiratory tract [2]. Overstimulation of this protective reflex leads to coughing spasms, which can be debilitating physically, socially, and psychologically. Secondary complications can include ruptured blood vessels, rib fractures, urinary incontinence, headaches, dizziness, musculoskeletal pain, hoarseness, insomnia, and exhaustion. Patients with chronic cough may also experience psychosocial complications including significant social withdrawal and self-consciousness [2]. People in close proximity to these patients may experience nosemaphobia, or fear of becoming ill, due to the uncertainty of the etiology of their cough.

NCC typically presents with a dry cough, often in spasms, preceded by a tickle or urge-to-cough. It is a reflex cough, initiated by the stimulation of sensory afferents, which have neural connections that elicit a cognitive drive or urge-to-cough [3]. Characteristic to NCC are multiple triggers. Talking and laughing, eating and drinking, cold air or change in humidity, and odors tend to trigger a tickle and subsequent coughing episode. Stimulation of the external auditory canal has been known to elicit a cough reflex in some known as Arnold's nerve reflex, mediated by the auricular branch of the vagus nerve.

This case report outlines three patients with chronic cough. Attempted treatments included a trigger reduction approach, laryngopharyngeal reflux control with a plant-based Mediterranean style diet, and medications for NCC, all of which failed to resolve cough symptoms [1, 4, 5]. Examination revealed one strand of hair within the ear canal with one end on the tympanic membrane and another on the anterior canal floor within the potential distribution of Arnold's nerve reflex sensory afferents. Removal of the hair resulted in substantial improvement or complete resolution of their cough.

## 2. Case Report

### 2.1. Case 1

A 59-year-old male presented with a nonproductive, dry cough for over a year. His cough was preceded by a tickling or itching sensation in his throat. Cough would occur in spasms. His self-reported triggers included talking, laughing, heat, smoke, and certain perfumes. He tried a nasal steroid spray, nasal irrigation, and reflux medication with minimal improvement of symptoms. His medications included losartan which was discontinued two weeks prior to his visit with no change in his cough. On physical examination, the patient was noted to have a foreign body in his left ear, notably two strands of hair lying on the left tympanic membrane. The hair was removed under direct vision, and the left ear canal had an otherwise normal appearance. Laryngoscopy revealed mild supraglottic hyperfunction, left vocal fold paresis, asymmetric vibration, slight anterior gap, and bilateral mild decrease in vibration, suggestive of a mild vocal fold neuropathy and neurogenic cough.

At his one month follow-up visit, he reported 30% improvement in cough symptoms. He was instructed on Mediterranean style dietary changes with alkaline water for improvement of laryngopharyngeal reflux. At his follow-up, three months after the initial visit, the patient reported continued improvement in cough. He continued to use nasal saline irrigations and diet adjustments for maintenance of persistent chronic cough symptoms. He was not interested in trying amitriptyline.

### 2.2. Case 2

A 70-year-old woman with history of chronic rhinitis and chronic cough presented with complaint of pressure and feeling of water in her right ear. Coughing fits were triggered by a tickle in her throat and would come on randomly. On physical examination, the patient was noted to have a foreign body in her right ear and 5-6 strands of hair were removed from around and leaning on the tympanic membrane. The hair stands were removed relieving her feeling of fullness. In further conversation after her visit, she revealed that her chronic coughing symptoms completely resolved after the hair removal. During follow-up with her audiologist a year later, she was noted to have hair in her right ear again, which was removed. Her chronic rhinitis persisted with improvement of cough symptoms.

### 2.3. Case 3

**Figure 1:**
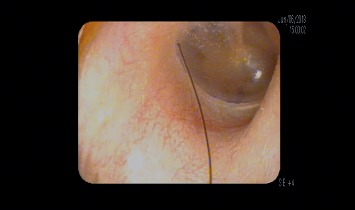
Hair resting on the tympanic membrane.

A 51-year-old man presented with voice problems and coughing spasms for over 1.5 months. His cough began before his voice changed. He felt a tickle in his throat that made him cough. Treatment by his primary medical doctor included antibiotics, an inhaled steroid, and reflux medication, which resulted in no change and were discontinued. During coughing spasms, his eyes teared, his voice became strained, and he became nauseous. He also reported coughing about two minutes after eating. The cough was not triggered by laughing, talking, drinking liquids, or being exposed to cold or certain odors. On physical examination, the patient's left ear canal had a strand of hair lying on the tympanic membrane (Figure 1). The foreign body was removed. Laryngoscopy revealed a large hemorrhagic polyp on the left midvocal fold with multiple bilateral ectasias. These findings were likely secondary to the trauma from coughing spasms. The patient noted a cessation of the tickle sensation from the neck to the throat immediately after removal of the foreign body. The patient did not follow up after initial presentation, presumably due to resolution of coughing symptoms.

## 3. Discussion

Chronic cough is a frequently encountered condition that accounts for 30 million office visits per year. Patients cumulatively spend $600 million on prescription and over-the-counter medications annually for treatment of chronic cough [6]. Over the last decade, further understanding of the sensory and motor pathways of the vagus nerve has highlighted NCC as a major cause of chronic cough in the majority of people who are not responsive, or minimally responsive to treatment of allergy, asthma, sinonasal conditions, and reflux, previously thought to be responsible for over 95% of chronic cough cases [7]. Patients with NCC often lack sinus symptoms and findings such as pus, nasal polyps, or severe obstruction. Many of these patients have normal CXR, CT chest, and pulmonary function testing as well as a history of poor response to pulmonary inhalers and other medications, typically including multiple courses of antibiotics. A recent study highlighted the frequent degree of misdiagnosis of asthma. One-third of the participants in the study were found to have no evidence of asthma during serial evaluations after the tapering of their asthma medications. This misdiagnosis prevents physicians from investigating the true cause of the patient's symptoms [8].

The chronic cough hypersensitivity model suggests that peripheral inputs, such as viruses, allergens, and air pollutants, can upregulate host cough responses and prompt phenotypic switches in the sensory neurons, leading to a state of hyperresponsiveness. Sensory neurons can show an increased expression of transient receptor potential (TRP) ion channels [9]. The three cases above illustrate a cough reflex likely elicited from stimulation of the somatic sensory portion of the auricular branch of the vagus nerve (ABVN) innervating the external auditory canal. The auricular branch originates from the superior ganglion of the vagus nerve within the jugular foramen, and it continues between the internal jugular vein and the bony wall of the jugular foramen approaching the mastoid canaliculus. The course of the ABVN parallels the tympanic branch of the glossopharyngeal nerve, with nerve fibers connecting the two. The precise distribution of the fine distal branches of the nerve is not well described. Individual differences in anatomy may account for variability found in patients tested for the reflex response [10]. While this reflex has only been noted in a small fraction of patients in the past, a recent study found a higher prevalence of Arnold's nerve reflex sensitivity in patients with chronic cough compared to healthy individuals [11]. This finding supports the notion that vagal hypersensitivity may play a larger role in chronic cough than previously expected. Vibration of the tympanic membrane due to the hair on the surface may also have contributed to the overstimulation of the ear canal as noted in the patients above.

All three patients in the cases above experienced an improvement of their cough symptoms after removal of hair resting on the tympanic membrane and their external auditory canal. The removal of the foreign material from the ear subsided the urge-to-cough and removed the trigger of the afferent sensory stimulus. All of the patients were instructed to place cotton balls in their ear canals during haircuts. While it is not a common etiology, foreign body in the ear should be considered in patients presenting with cough. Self ear cleaning is a common practice, and improper technique poses a risk for injury, inflammation, or foreign body complications within the ear [12]. Educating patients on safe methods for ear cleaning is also an important preventative measure. Patients' symptoms can be easily resolved as long as the offending agent is recognized by the physician during the physical examination. We propose this condition be entitled “Oto-tricho-tussia,” which describes the location of a hair within the ear stimulating Arnold's nerve, a branch of the vagus, generating a cough.
